# The association between self-efficacy, perceived social support, and family resilience in patients undergoing hemodialysis: a cross-sectional study

**DOI:** 10.1186/s12882-024-03629-4

**Published:** 2024-06-25

**Authors:** Farzaneh Safi, Hossein Namdar Areshtanab, Mansour Ghafourifard, Hossein Ebrahimi

**Affiliations:** 1 Department of Psychiatric Nursing, Faculty of Nursing and Midwifery, Tabriz University of Medical Sciences, Tabriz, RN Iran; 2 Medical Education Research Center, Health Management and Safety Promotion Research Institute, Tabriz University of Medical Sciences, Tabriz, Iran; 3 Department of Medical Surgical Nursing, Faculty of Nursing and Midwifery, Tabriz University of Medical Sciences, Tabriz, Iran

**Keywords:** Self-efficacy, Family resilience, Social support, Chronic kidney disease, Hemodialysis

## Abstract

**Background:**

Self-efficacy of patients on hemodialysis is considered a main component of the successful management of chronic kidney diseases. The self-efficacy of these patients may be influenced by many individual and social factors. This study aimed to assess the association between perceived self-efficacy and social support by patients on hemodialysis treatment and the resilience of their families.

**Methods:**

This cross-sectional study was conducted on 183 patients and 183 families of hemodialysis patients in the largest hemodialysis center in northwest of Iran. Data was collected from July to December 2021 using chronic kidney disease self-efficacy, multidimensional perceived social support (MSPSS), and the Walsh family resilience questionnaire (WFRQ). The collected data were analyzed by SPSS software using descriptive and inferential statistical tests.

**Results:**

The findings showed that the mean score of patients’ self-efficacy was 171.63 ± 38.19 in a possible range of 25 to 250. Moreover, the mean score of perceived social support was 62.12 ± 16.12 in a possible range of 7 to 84. The mean total score of family resilience was 119.08 ± 26.20 in a possible range of 32 to 84. Also, the results of the study showed a positive and significant relationship between the self-efficacy of patients with their perceived social support and the resilience of their families (*p* < 0.01).

**Conclusion:**

The results of the study showed that there is a significant relationship between patient self-efficacy and family resilience and social support received in chronic kidney patients undergoing hemodialysis. Therefore, it is suggested to consider practical strategies in the field of family resilience and social support to improve patients’ self-efficacy.

## Background

The final stage of kidney failure is a chronic, irreversible, and life-threatening disease [1]. Chronic kidney disease is associated with many biochemical and physiological changes and may have important adverse consequences including increased risk of cardiovascular events, long-term care, and expensive replacement treatments leading to reduced quality of life and early death [2, 3]. Hemodialysis (HD) is the most common renal replacement therapy in the world [4]. The main purpose of hemodialysis is to maintain the balance of the fluid in the inside and outside of the cells, which is one of the characteristics of kidney function. This modality improves the patients’ health and increases their survival, but it does not change the course of the disease and does not completely replace the function of the kidneys [5]. According to the WHO report, kidney and urinary tract diseases cause about 850 thousand deaths and disable 115 million people annually [6]. The average growth of this disease in the last five years was about 8% [7]. Around the world, approximately 600 million people are suffering from chronic kidney disease and more than 60 thousand people die from this disease annually. In Iran, the growth of this disease is higher than the global average and its prevalence is between 1200 and 1600 people annually [8]. It is estimated that the number of patients with end-stage kidney disease (ESKD) who need renal replacement therapy will increase from 2.6 million people in 2010 to 5.4 million people in 2030 worldwide [9].

Treatment with hemodialysis is a condition that severely affects the patients’ personal, family, work, and social life [10]. Although this modality helps people to continue their lives, but patients should adhere to some strict medical recommendations such as food restrictions and balanced fluid intake, regular use of medicines, and regular visits for treatment [11]. Moreover, they need some special and continuous training on life style [12]. Hemodialysis limits a person’s physical, emotional and social functioning and causes dissatisfaction with life and a decrease in quality of life. To improve the quality of life of hemodialysis patients, ensuring their adherence to treatment and guiding them to achieve healthy lifestyle behaviors is very important [13]. Patients’ quality of care could be influenced by individual factors such as self-efficacy and social factors such as family resilience and social support received [14–16].

Self-efficacy is one of the most important individual factors of hemodialysis patients [17]. Self-efficacy means the belief that a person has in himself/herself to perform a certain behavior successfully and expect positive outcomes [18]. Self-efficacy can improve the cognitive and emotional performance of patients, reduce mortality and hospitalization, and finally improve the patients’ adherence to treatment [19]. Identifying and planning to improve the patient’s self-efficacy can lead to improving self-management, increasing life expectancy, and modifying health behaviors [20]. Several studies have shown that patients with better self-efficacy experience a high quality of life than patients with lower self-efficacy. Additionally, the better self-efficacy improves the patients’ daily activities and their adherence to treatment [17, 21, 22]. Moreover, a study in Indonesia showed that low self-efficacy causes non-compliance and unfollow-up of treatment in patients with chronic kidney disease [17]. Moreover, according to a study in Iran, most of the patients on hemodialysis have worse self-efficacy. Therefore, the identification of factors related to the self-efficacy of these patients seems necessary [23].

As a new concept, resilience could play an active role in disease management and clinical outcomes in patients on HD [10]. A resilient person processes the unfortunate situation in a more positive way and considers himself/herself capable of facing it [24]. In fact, resilience is an important factor that protects a person from the effects of traumatic situations. It is a personality trait that increases individual adaptation and has a positive effect on adaptation and successful coping with traumatic situations [25]. Caregivers and patients’ family play an important role in the management of many chronic diseases [26]. Since caregivers have roles and responsibilities in all stages of disease from diagnosis to discharge and they provide home care, it increases their care burden. Problems experienced by caregivers, which increase their burden of caregiving and, accordingly, their quality of life worsens and complicates the situation [27]. Many serious psychological and physiological problems can occur in caregivers whose burden of care increases and their quality of life deteriorates [28]. Nowadays, the researchers pay more attention to the role of family resilience for better management of chronic diseases such as CKD. It is defined as a family’s capacity to endure and recover from adversity and to become more resourceful and stronger [29]. Each family may face some problems, challenges, and adversity at any stage of life [30]. When families face a crisis or stressor, they try to balance their needs and capacity. How families make changes depends on the skills and resources available to them to overcome the crisis, which creates different levels of resilience [31]. Good relationships between family members are significantly associated with improved emotional well-being and better quality of life [25].

On the other hand, various factors can play a role in the consequences of chronic diseases such as kidney failure, which includes social support [32]. Social support includes receiving formal and informal emotional, instrumental and informational help, and patients who get high scores in social support are more likely to cope better with psychological stressors [33]. High levels of social support promote psychological resilience and positive psychological outcomes for maintaining a person’s physical and mental health [34]. Recent studies show that social support, including support from spouses, family members, and friends, is significantly related to the health of chronic patients [32] Family support is very important, so that patients have self-confidence and can be under self-management, which may lead to more self-efficacy [35]. In our country, the social support is done mainly by patient’s family and other significant persons who have an intimate relationship with patients. Moreover, there are some nongovernmental organizations such as Iranian kidney foundation that support the patients with CKD [36].

One of the primary obstacles for individuals with chronic illnesses is self-management. It is evident that as self-confidence grows, individuals will engage in better self-care, leading to improved disease management and living conditions over the course of their lives, ultimately enhancing the quality of life for patients. As patients’ self-confidence increases, the effectiveness of medical and nursing care also grows. Nurses can have a substantial impact on enhancing patients’ self-confidence by empowering their families and caregivers.

Based on the literature review, there is little research on family resilience among patients undergoing hemodialysis. Moreover, no study was found regarding the association of self-efficacy, social support, and family resilience in patients with CKD. This study aimed to assess the relationship between self-efficacy, family resilience and social support in patients undergoing hemodialysis.

## Materials and methods

### Study design

The present study was a descriptive correlational study that was conducted from July to December 2021 at the largest hemodialysis center in northwest of Iran.

### Participants and sampling

Sampling was done using a simple random sampling from patients on hemodialysis and their families who were referred to the Imam Reza Teaching Hospital in Tabriz, Iran. We used a Randomizer software for generation of random numbers. The inclusion criteria for the patients included: patients aged 18–85 years with willingness to participate in the study, at least 6 months being treated with maintenance hemodialysis, and undergoing HD at least twice a week. The inclusion criteria for the family included: the patient’s family who lives with the patient and takes care of him/her. Exclusion criteria for patients and families included unwillingness of themselves or the patient’s family to participate in the study and incomplete completion of study questionnaires.

The population of patients undergoing hemodialysis in Imam Reza teaching hospital was 350 people. The sample size was determined using Krejcie and Morgan table. According to the Krejcie and Morgan, the table provides the recommended sample size for different population sizes, ranging from 10 to 1,000,000. Researchers can use the table to determine the minimum sample size required for their study based on the known or estimated population size. Accordingly, since there were 350 patients with CKD undergoing hemodialysis in the studied dialysis center, a total of 183 patients and families were determined as sample size using the Krejcie and Morgan Table [37].

### Instruments

Data was collected using demographic information and three questionnaires including the family resilience index, self-efficacy of chronic kidney patients, and multidimensional perceived social support (MSPSS).

Demographic data included information related to patients’ age, gender, education, marital status, occupation, and economic status, place of residence, living with family, and relationship with the patient.

The Walsh Family Resilience Questionnaire (WFRQ) was used to assess the family resilience [38]. This is a 32-item questionnaire scored on a 5-point Likert scale (1 = rarely to 5 = usually). The questionnaire has three subscales: 1- belief systems 2- organizational patterns and 3- communication processes. The minimum score in this index is 32 and the maximum score is 160. Higher scores indicate greater family resilience. The validity and reliability of this tool were investigated by Dadashi et al. in Iran and the Cronbach’s alpha coefficient of the questionnaire was reported to be higher than 0.70 [39]. In this study, the reliability of the tool was recalculated using Cronbach’s alpha of 0.97.

The CKD self-efficacy (CKD-SE) questionnaire was used to measure the self-efficacy of patients undergoing HD. The questionnaire includes 25 items with four subscales of autonomy, self-integration, problem-solving, and seeking social support. According to the patients’ perception, the answers to each item ranged from completely uncertain (score 0) to the highest degree of confidence (score 10). The higher scores indicate greater self-efficacy. The minimum and maximum total score of the tool is 0 and 250, respectively. The validity and reliability of the Persian version of this tool have been investigated by Lacke et al. [23]. It’s validity has been evaluated using content validity and the reliability has been reported using Cronbach’s alpha of 0.95, which indicates the acceptable level of reliability. In this study, the content validity was measured by the comments of 10 members of the nursing faculty of Tabriz University of Medical Sciences, and the reliability of the tool was calculated using Cronbach’s alpha of 0.97.

The Multidimensional Scale of Perceived Social Support (MSPSS) scale was used to measure the social support perceived by HD patients from three sources: family, friends, & significant other. It is a 12-item questionnaire and the answers to each item ranged from 1 (strongly disagree) to 7 (strongly agree) [40]. The minimum and maximum score is 12 and 84, respectively. A higher score indicates greater perceived social support. The validity and reliability of the Persian version of this tool were assessed by Besharat et al. [41]. , and the Cronbach’s alpha coefficient for the whole scale and three sub-items of the three sub-scales of family, social, and friends support were calculated as 0.91, 0.87, 0.83 and 0.89, respectively. In this study, the content validity was measured by using the comments of 10 members of the nursing faculty of Tabriz University of Medical Sciences, and the reliability of the instrument was calculated using Cronbach’s alpha of 0.96.

### Data collection

Permission to conduct the study was obtained from the regional ethics committee of Tabriz University of Medical Sciences (IR.TBZMED.REC.1400.1120). The objective of the study was explained to the patients and families. Moreover, they were assured of voluntary participation in the study, the confidentiality of information, and the right to withdraw from the study. The social support and self-efficacy questionnaire was completed by the patients and the family resilience questionnaire was completed by the patients’ families (the main caregiver of the patient who has the most contact with the patient). The Helsinki ethical statement was observed in patients and families.

### Data analysis

The collected were analyzed using SPSS version 22 software. Kolmogorov-Smirnov test was used to determine the normal distribution of data and Spearman’s correlation test was used to check the relationship between variables. The significant level was considered as 0.05.

## Results

A total of 183 patients and 183 families participated in this study. Among patients, 114 were women and 69 were men (Table 1). The average age of the patients participating in this study was 62.8 ± 11 years and the average age of the caregivers was 48.6 ± 11.8 years (Tables 1 and 2). About, 67% of the patients were married and 79% of them lived in the city (Table 1).

**Table 1:** Distribution of demographic characteristics of patients

Variable			*N*(%)
Age (Yrs.)	< 30		3(2)
	30-40		6(3)
	41-50		17(9)
	51-60		37(20)
	61-70		78(43)
	>70		42(23)
Sex	Female		114(62)
	Male		69(38)
Level of Education	Undergraduate		107(58)
	High school diploma		56(31)
	College		20(11)
Marital status	Single		7(4)
	Married		123(67)
	Divorce		5(3)
	Widow		48(26)
Job	Housewife		97(53)
	employed		64(35)
	unemployed		22(12)
Economic status	Expenditure is equal to income		59(32)
	Expenditure is less than income		40(22)
	Expenditure is more than income		84(46)
Resident	Urban		146(80)
	Rural		37(20)
living situation	Alone		36(20)
	Wife, Child		115(63)
	Parents		6(3)
	Others		26(14)
Relationship of the family member with the patient	Mother		6(3)
	Wife		95(52)
	Child		82(45)
	Min	Max	M(SD)
Patient age	25	83	62.8±11
Number of children	2	4	3.69±0.52

**Table 2:** Distribution of demographic characteristics family of patients

Variable			*N*(%)
Sex	Female		102(56)
	Male		81(44)
Level of Education	Undergraduate		65(35)
	High school diploma		70(38)
	College		48(27)
Marital status	Single		17(9)
	Married		161(88)
	Divorce		2(1)
	Widow		2(2)
Job	Housewife		77(42)
	employed		96(53)
	unemployed		10(5)
Economic status	Expenditure is equal to income		79(43)
	Expenditure is less than income		43(23)
	Expenditure is more than income		61(34)
Difficulty in paying treatment costs	Yes		72(39)
	No		111(61)
Relationship of the family member with the patient	Mother		6(3)
	Wife		95(52)
	Child		82(45)
	Min	Max	M(SD)
Caregiver age	19	76	48.6±11.8

The total score of patient family resilience and its dimensions including belief system, organizational model, and communication process are shown in Table 3. The highest score in the (WFRQ) belonged to the communication process subscale (37.69 ± 8.54) and the lowest score belonged to the organizational model subscale (32.56 ± 7.23) (Table 3).

**Table 3:** Mean and SD of family resilience scores

	Achievable score	Min	Max	M(SD)	Median	Quartil1	Quartil3
Belief systems	13-65	22	64	48.82±11.37	54	39	57
Organizational patterns	9-45	14	65	32.56±7.23	35	26	38
Communication processes	10-50	17	49	37.69±8.54	42	32	45
The total score of resilience	32-160	56	153	119.08±26.20	129	91	140

The total self-efficacy score of patients undergoing hemodialysis and its subscales including independence, organization, problem-solving, and seeking social support are shown in Table 4. The highest score belonged autonomy subscale (58.62 ± 12.63), and the lowest score to the seeking social support subscale (29.29 ± 6.93) (Table 4).

**Table 4:** Mean and SD of the self-efficacy of the patients

	Achievable score	Min	Max	M(SD)	Median	Quartil1	Quartil3
Autonomy	8-80	27	77	58.62±12.63	61	48	70
Self-integration	7-70	21	66	46.97±12.46	48	36	59
Problem -solving	6-60	19	56	36.75±10.76	38	26	46
Seeking social support	4-40	11	40	29.29±6.93	31	26	34
The total score of self-efficacy	25-250	81	233	171.63±38.19	169	134	205

The total score of perceived social support of hemodialysis patients and its subscales including family, friends, and social are shown in Table 5. The highest score on the questionnaire belonged to the social subscale (21.64 ± 5.63) and the lowest score to the friends subscale (19.28 ± 6.81) (Table 5).

The results of the Kolmogorov-Smirnov test showed that the variables of family resilience (Z = 0.19, *p* < 0.01), self-efficacy (Z = 140, *p* < 0.05), and social support received (Z = 0.179, *p* < 0.01) do not have a normal distribution. Therefore, Spearman’s rank correlation test was used to check the relationship between study variables. Based on the results of the Spearman correlation test, there was a positive and significant relationship between each domain of the perceived social support questionnaire with each domain of the chronic kidney disease self-efficacy questionnaire (*p* < 0.01). Moreover, the results showed that there was a positive and significant relationship between the domains of the perceived social support questionnaire and each domain of the family resilience questionnaire (*p* < 0.01). According to the results, each domain of the chronic kidney disease self-efficacy questionnaire showed a positive and significant relationship with the single domains of the family resilience questionnaire (*p* < 0.01) (Table 6).

**Table 5:** Mean and SD of the perceived social support of the patients

	Achievable score	Min	Max	M(SD)	Median	Quartil1	Quartil3
Family	4-28	6	27	21.19±5.35	23	18	25
Friends	4-28	4	28	19.28±6.81	22	13	26
Social	4-28	6	28	21.64±5.63	24	18	26
Total score	7-84	20	83	62.12±16.12	69	49	76

## Discussion

The results showed that there was a positive and significant relationship between all domains of the perceived social support questionnaire (family, friends, and socially influential people) with all domains of the chronic kidney disease self-efficacy questionnaire (independence, organization, problem-solving, and seeking social support). In line with our study, Kiajamali et al. [42] found a positive and significant relationship between social support and self-efficacy in hemodialysis patients (*r* = 0.592, *p* < 0.001). Also, Mollaoglu et al. (2006) reported that hemodialysis patients who perceived higher levels of social support were more likely to have higher levels of self-care [43]. Both studies were consistent with the findings of the present study. However, Yang et al. (2020) conducted a study on patients with mental disorders and found no significant relationship between social support and self-efficacy in these patients [44]. The reason for this difference can be the disruption of interpersonal capabilities in psychiatric disorders. These patients chronically suffer from a low level of self-confidence, self-efficacy, self-concept, and self-awareness. Therefore, even with favorable social support, the level of self-efficacy may not change [45]. Social support can affect a person’s self-efficacy in various ways, such as giving encouragement and punishments while performing various daily activities [46]. This issue is felt more in chronic patients, especially hemodialysis. Due to the long term period of hemodialysis treatment, the patient faces some challenges in daily activities, changes in lifestyle, and various needs. They need to adapt to these limitations [47]. Understanding social support is effective in adapting people to the disease and its complications.

Yu-ChiChen et al. (2018) concluded that social support in patients with chronic kidney disease is related to self-management and self-efficacy. According to their findings, nurses should develop patient self-management programs with the participation of patients’ families and adopt a systemic approach to help chronic kidney patients improve their self-management behaviors in order to increase their self-efficacy [48]. Perceived social support refers to the extent to which a person considers his social system, which includes family, friends, and important social persons, to be supportive in terms of emotional resources, information, and companionship. Thus, this construct reflects how much a person perceives that they can rely on their social network when they need help [49]. If a person sees himself supported in the three important aspects of social support (family, friends, and other significant persons), the person gains more confidence in his/her abilities to control his motivation, behavior, and social environment [50, 51]. In a study in China, Song et al. [52]. , investigated social support, sense of coherence, and self-management in hemodialysis patients. They found that social support is effective in self-management and a sense of coherence in HD patients. They reported a moderate level of social support in the patients. However, the level of social support in our study was higher than average, which can be attributed to the social and cultural differences of the two communities, the difference in the tools used in two studies, and the difference in the sample size of the studies.

Also, the results showed that there is a positive relationship between each domain of the perceived social support questionnaire (family, friends, and socially influential people) with each domain of the patients’ family resilience questionnaire (belief system, organizational model, and communication process). In a recent study on hemodialysis patients, Qiu et al. [53] showed that family resilience and social support increase potential psychological resilience. Interventions to improve family resilience and social support are beneficial to promote the psychological resilience of hemodialysis patients. In another study on chronic cancer patients, Chen et al. (2021) found a significant relationship between the perceived social support and the resilience of the patients’ families (*r* = 0.24–0.32, *p* < 0.01), which was in line with the present study [54]. This issue is more tangible in dialysis patients due to the chronic process of the disease. Hemodialysis treatment preserves the life of patients with end-stage kidney disease but does not prevent emotional suffering related to chronic stress related to disease burden, dialysis treatment, functional limit, and fear of death. Therefore, they require social support to cope with these challenges [53]. Family, friends, and social influencers are among the most important factors influencing each person’s support. A long relationship with these people affects the belief system, organizational pattern, and communication process of each person. The greater the resilience of the family, the more support is given to the individual. Hoang et al. [55] investigated the social support in hemodialysis patients and found that the mean score of social support was 58.60 ± 22.30 in a possible range of 0 to 100. This finding is not in line with our results where the mean score of social support was at higher level. It seems that this difference could be explained by the difference in the culture of hemodialysis patients in two studies.

According to the results, there was a positive and significant correlation between the domains of the chronic kidney disease self-efficacy questionnaire (independence, organization, problem-solving, and seeking social support) with all domains of the patient’s family resilience questionnaire (belief system, organizational model and process). Isnaini et al. (2021) studied hemodialysis patients and found a strong relationship between family support and patient self-efficacy so patients with chronic kidney disease need family support [35]. Yin et al. (2022) investigated chronic cancer patients and reported that the self-efficacy of these patients has a significant relationship with the resilience of the patients’ families (*p* < 0.001), which was in line with the present study [56]. Zhang et al. (2023) investigated chronic stroke patients and found a significant relationship between self-efficacy and resilience of patients’ families (*p* < 0.001) [22]. It can be concluded that the subscales of self-efficacy which include independence, organization, problem-solving, and seeking social support, can be affected by the belief system, organizational model, and communication process in each family. This issue is more important in chronic diseases, especially hemodialysis, where the relationship between the family and the patient is high. Families are frequently in contact with the patient, and the patient is dependent on the family support for coping with this chronic disease [57]. The more resilient in patients’ family, the less tension occurs between them. But in families with lower resilience, tension may arise and the family sometimes exposes the burden of care to the patient. Over time, this issue causes frustration, depression, decreased self-confidence, and decreased self-efficacy in the patient [58, 59]. These complications may even cause the patient to have mental problems over time. The sensitivity of this issue is more important when we understand that patients are exposed to mental disorders due to their invasive measures, devices, and new technologies, and the family plays an essential role in reducing these tensions [60, 61]. Therefore, in future studies, it is suggested to investigate the effect of resilience training in reducing stress in hemodialysis patients and their families. Improving family resilience in this field can influence on social support and self-efficacy of dialysis patients. Therefore, it is suggested to consider clinical trial studies to assess the casual effect of family resilience on patient’s self-efficacy.

**Table 6:** Correlation between self-efficacy and perceived social support of patients and family resilience

	Self-efficacy					Family resilience				Perceived social support			
	1	2	3	4	5	6	7	8	9	10	11	12	13
1- Autonomy	1												
2-Self-integration	0.878^**^	1											
3-Problem -solving	0.643^*^	0.742^*^	1										
4-Seeking social support	0.759^*^	0.689^*^	0.523^*^	1									
5-The total self-efficacy	0.936^*^	0.951^**^	0.831^*^	0.805^*^	1								
6-Belief systems	0.720^**^	0.682^*^	0.598^*^	0.748^*^	0.765^*^	1							
7-Organizational patterns	0.638^*^	0.606^*^	0.561^**^	0.710^*^	0.696^**^	0.884^*^	1						
8-Communication processes	0.657^*^	0.641^*^	0.603^*^	0.731^*^	0.729^*^	0.926^*^	0.863^**^	1					
9-The total resilience	0.703^*^	0.672^*^	0.611^*^	0.759^*^	0.762^*^	0.980^*^	0.941^*^	0.966^*^	1				
10-Family	0.689^**^	0.594^*^	0.396^*^	0.809^*^	0.680^*^	0.613^*^	0.554^*^	0.590^*^	0.611^*^	1			
11-Friends	0.591^*^	0.555^*^	0.594^*^	0.536^*^	0.641^*^	0.575^*^	0.532^*^	0.587^*^	0.588^*^	0.632^*^	1		
12-Social	0.706^*^	0.583^*^	0.388^*^	0.788^*^	0.676^*^	0.637^*^	0.552^*^	0.612^**^	0.629^*^	0.915^*^	0.681^**^	1	
13-Total social support	0.725^*^	0.635^**^	0.517^*^	0.770^*^	0.732^*^	0.669^**^	0.601^*^	0.658^*^	0.671^*^	0.913^*^	0.869^**^	0.940^*^	1

*P < 0.01 using the Spearman’s rank correlation test

** *P* < 0.05 using the Spearman’s rank correlation test

## Limitations

One of the limitations of the research is the limited number of samples that refer to the treatment center. Moreover, it is worth mentioning that one of the most important limitations of the study was the deterioration of patients during dialysis, the complications of lowering blood pressure, the impatience of patients due to their old age, which made it difficult to complete the questionnaire. Therefore, we completed the questionnaire within 1 h of beginning the dialysis treatment. This study was done in a single dialysis center in Iran, so it is recommended to conduct others studies in the multicenter dialysis units. This study was done only on patients undergoing hemodialysis, it is recommended to do the similar study on patients with other renal replacement therapies including peritoneal dialysis or kidney transplantation. The comparison between hemodialysis, peritoneal dialysis, and kidney transplantation could provide a deep understanding of the concepts.

## Conclusion

The results of the study showed that there was a significant relationship between family resilience, patient self-efficacy, and social support received in chronic kidney patients undergoing hemodialysis treatment. Family, friends, and community are the most important sources of social support that influence on independence, organizing, solving problems, and seeking social support for patients. Health care providers should encourage patients’ family to collaborate with health care provider and participate in the shared decision making on patients’ needs. Moreover, they should be encouraged to support the patients emotionally. Improving family resilience in this field can influence on social support and self-efficacy of dialysis patients. Therefore, it is suggested to consider clinical trial studies to assess the casual effect of family resilience on patient’s self-efficacy.

## Data Availability

The dataset supporting the conclusions of this article is included within this article.
